# Prehospital Emergency Nurse competence and pain management in patients with acute abdominal pain: a retrospective observational study

**DOI:** 10.1186/s12873-026-01707-4

**Published:** 2026-07-29

**Authors:** Rasmus Bjerén, Carl Magnusson, Johan Herlitz, Martin Flores Bjurström, Magnus Hagiwara, Denise Bäckström

**Affiliations:** 1 Center for Research and Development, Region Gävleborg, Gävle, Sweden; 2 Department of Biomedical and Clinical Sciences, Linköping University, Linköping, Sweden; 3 Department of Prehospital Emergency Care, Sahlgrenska University Hospital, Gothenburg, Sweden; 4 Center for Prehospital Research, Faculty of Caring Science, Work Life and Social Welfare, University of Borås, Borås, Sweden; 5 Department of Surgical Sciences, Clinical Pain Research, Uppsala University, Uppsala, Sweden

**Keywords:** Emergency medical services, Abdominal pain, Analgesia, Pain measurement, Emergency nursing, Prehospital care

## Abstract

**Background:**

Pain is common in emergency medical services, and abdominal pain is among the most frequent complaints. This study examined whether documented pain assessment, analgesic treatment, reassessment, and documented end-of-encounter pain status in patients with acute abdominal pain differed by clinician competence (Prehospital Emergency Nurse [PEN] vs. non-PEN), patient sex, age, and prehospital interval.

**Methods:**

This retrospective observational study was a secondary analysis of adult patients with acute abdominal pain in a previously described cohort. Electronic patient records were reviewed in full, including Numerical Rating Scale (NRS) scores and free-text pain descriptions. Primary outcomes were documented pain assessment, analgesia administration, and any documented reassessment. Secondary outcomes were documented NRS use, analgesia among patients with moderate–severe pain, reassessment after analgesia, and mild pain at the last documented assessment. Age and prehospital interval were dichotomized at the median. Mann–Whitney U and Fisher’s exact tests were used, with *p* < 0.01 considered statistically significant.

**Results:**

Of 840 sampled records, 816 were included. Median age was 64 years (IQR 41–79), and median prehospital interval was 38 minutes (IQR 27–60). Compared with non-PEN encounters, PEN encounters had higher rates of pain assessment (67% vs. 53%, *p* = 0.003), analgesia administration (50% vs. 37%, *p* = 0.005), and reassessment (31% vs. 17%, *p* < 0.001). Among patients with moderate–severe pain, analgesia was more frequent in PEN encounters (81% vs. 58%, *p* < 0.001). Mild pain at the last documented assessment was also more common in PEN encounters (23% vs. 8%, *p* < 0.001). Younger patients and cases with longer prehospital intervals had higher rates of pain assessment, NRS use, analgesia administration, and reassessment, but not mild pain at last assessment.

**Conclusions:**

The PEN encounters showed higher rates of documented pain assessment, analgesia administration, and reassessment, with a higher proportion of patients having mild pain at the last documented assessment. Differences related to age and prehospital interval were mainly limited to process measures. Further research is needed to clarify whether clinician competence and care processes translate into improved pain relief.

## Background

Pain is experienced by approximately half of the patients in prehospital care [1–3], most often with a moderate or severe intensity [4, 5]. One of the most common presenting complaints is acute abdominal pain (AAP), constituting around 22% of the pain-related assignments [4]. Provision of effective pain management is recognized as fundamental for high-quality care [6] and a human right [7]. Thus, it has been suggested that all Emergency Medical Services (EMS) patients should be considered potential candidates for analgesia [8], and most prehospital guidelines recommend prompt treatment of moderate and severe pain [9]. A documented pain assessment has been linked to a higher likelihood of analgesic treatment [4]. As pain tends to be underestimated by clinicians [10], it should preferably be assessed using patient self-report and a validated scale such as the Numerical Rating Scale (NRS), where zero represents no pain, 1–3 mild, 4–6 moderate and 7–10 severe pain [11]. While a reduction of one to two NRS units has been described as a clinically meaningful improvement [12], poor guideline adherence and substantial rates of pain undertreatment have been reported [3, 13, 14]. Unrelieved pain may contribute to unnecessary suffering, an increased risk of chronic pain [15], cardiovascular disease [16] and mortality [17]. In our previous AAP study, low levels of pain assessment, treatment and reassessment were identified and more patients than previously described had moderate to severe pain. Among patients who received adequate management, clinically significant pain relief, defined as a reduction of ≥ 2 NRS points, was achieved in four out of five patients, and severe pain declined from 70% to 22% [18].

Since previous research has reported differences in pain management according to factors such as sex [14], age [3], prehospital interval [13], and EMS clinician competence [3, 19, 20], it seems relevant to further explore our findings in relation to these factors. Sweden, like several other countries, has a nurse-based EMS system, with each ambulance crew including at least one registered nurse. Approximately two thirds of the nurses have a one-year master’s-level qualification as a Prehospital Emergency Nurse (PEN) [21]. Although PEN competence has been linked to higher self-reported professional competence [22], its association with prehospital pain management remains unexplored. At the same time, competence is a modifiable aspect of care delivery that could potentially affect care quality. Accordingly, this study aimed to examine whether documented pain assessment, analgesic treatment, reassessment, and documented end-of-encounter pain status differed by EMS clinician competence, patient sex, age, and prehospital interval among patients with acute abdominal pain.

## Methods

### Study design, setting and population

This retrospective observational secondary analysis was based on the same cohort of patients with acute abdominal pain as our previous investigation [18]. The study was performed in a Swedish region with approximately 300,000 inhabitants, including urban and rural areas. EMS was publicly funded and included 22 ambulances and one single-responder, primarily transporting patients to three hospitals [23]. Ambulances were staffed with at least one nurse, of whom 21% had PEN competence.

### Guidelines on pain management and documentation

Clinical practice was guided by local guidelines developed by the physician medical director and aligned with national recommendations from the Swedish Ambulance Physicians’ Network [24]. The guidelines advised documenting pain history and repeated pain scores, and recommended analgesia for NRS ≥ 4, although no lower limit was specified. The electronic patient record (EPR) included a non-mandatory pain score field alongside vital signs, multiple free-text fields for pain history, and a triage identifier for severe pain. Pre-authorized analgesics included paracetamol, esketamine, morphine, sufentanil, diclofenac, and alfentanil, administered through intravenous, peroral, intranasal, or intramuscular routes depending on drug and indication. A multimodal approach was permitted. The separate guideline for acute abdominal pain referred to the general pain management guideline regarding pain treatment, and physician consultation by phone was available at all times.

### Sampling and data collection

**Fig. 1:**
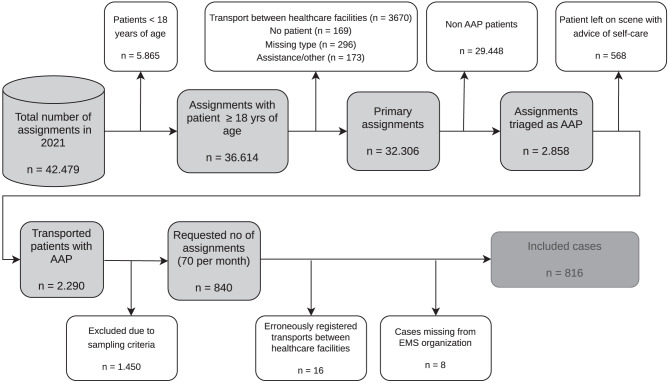
Overview of sampling and exclusion. As previously published in Bjerén et al. [18]

Data were obtained from EPRs within the regional EMS and abstracted by the first author, who had several years of clinical EMS experience and familiarity with the specific EPR system. To support consistent data abstraction, the abstraction procedure, variable definitions, and coding rules were tested and refined using a set of sample records not included in the final dataset. Eligible cases were primary assignments coded as abdominal pain by the EMS clinician. Exclusion criteria were age < 18 years, inter-facility transports, assistance to another ambulance, and assignments not resulting in transport. A structured convenience sampling strategy was used. To achieve an even distribution across seasons, the first 70 eligible records per month in 2021 were included, corresponding to a target sample of 840 cases (Fig. 1).

The EPRs were reviewed in full using a standardized abstraction form, with abstracted data entered into a pre-designed data sheet. Data abstraction followed predefined variable definitions and coding rules to reduce interpretive bias. Uncertainties were discussed within the research group, and coding rules were clarified when needed. As all data were abstracted by the first author, blinding to the study aim was not feasible, and interrater agreement could not be assessed. Pain assessments, pain scores, treatments, triage data, demographic data, EMS clinician competence, and time intervals were extracted. Pharmacological treatments were retrieved directly from the medication module in the EPR, whereas nonpharmacologic treatment was interpreted based on free-text descriptions. Prehospital interval was defined as the time from EMS arrival at the scene to hospital arrival, including on-scene and transport time. Data on EMS clinician competence were missing for 11 cases and prehospital interval data for 28 cases; these cases were excluded from the respective subgroup analyses.

### Outcomes

The outcomes of interest for this secondary analysis were defined a priori. The primary outcomes reflected key process measures of prehospital pain management and comprised the proportions of patients receiving pain assessment, analgesia administration, and any documented reassessment. Secondary outcomes included initial pain intensity, documented NRS use, analgesia among patients with moderate–severe pain, reassessment after analgesia, and mild pain at the last documented pain assessment.

Pain assessment was defined broadly as any documented evaluation of pain, including NRS scores, free-text descriptions, and a triage system identifier for severe pain. Use of NRS was analyzed separately as a specific form of quantitative pain assessment. Initial moderate–severe pain was defined as NRS ≥ 4 at the first documented pain assessment. When an NRS score was available, this value was used for classification; otherwise, qualitative free-text descriptions indicating moderate or severe pain were applied. If neither an NRS score nor a qualifying free-text description was available, the triage identifier for severe pain was considered as indicating severe pain, when used. Accordingly, classification of moderate–severe pain was restricted to cases with a documented initial pain assessment.

Any documented reassessment was defined as documentation indicating that pain had been reassessed after the initial assessment. When a reassessment NRS score was available, the last documented NRS value was used to describe pain intensity at reassessment. In some cases, reassessment was documented in free text without a clear resulting pain score, such as “morphine i.v. with good effect”. In these cases, pain was considered reassessed, but no resulting pain intensity was entered into the data sheet.

Mild pain at last assessment was defined as NRS ≤ 3 at the last documented NRS assessment. If no NRS score was available, qualitative documentation indicating mild pain was used. In cases without any documented pain assessment, mild pain could not be established and was therefore not classified as mild. This outcome reflects documented end-of-encounter pain status rather than change in pain intensity.

### Analysis

Outcomes were analyzed according to sex, age, EMS clinician competence, and prehospital interval. Age and prehospital interval were dichotomized at the median. EMS clinician competence was categorized as PEN or non-PEN. No formal sample size calculation was performed because this was a descriptive secondary analysis of a predefined cohort rather than a study designed to test a single primary hypothesis. For the same reason, and given the number of univariable comparisons performed, no formal adjustment for multiplicity was applied. Instead, a more conservative two-sided significance threshold of *p* < 0.01 was chosen to reduce the risk of type I error. The Mann-Whitney U test was used to compare ordinal and continuous variables, while Fisher’s exact test was used for binary variables. Categorical variables are presented as counts and percentages, and ordinal or continuous variables as medians with interquartile ranges (IQR). Statistical analyses were performed using SPSS Statistics version 28 (IBM Corp., Armonk, NY, USA).

The study is reported in accordance with the STROBE statement [25], and the reporting of the medical record review was guided by the methodological recommendations described by Gilbert et al. [26].

## Results

In all, 816 cases were included (Fig. 1). The median age was 64 years (IQR 41–79), and the median prehospital interval was 38 minutes (IQR 27–60). Fifty-six percent (*n* = 457) of the patients were female, and in 16% (*n* = 128) of cases the EMS clinician was a PEN. Baseline characteristics have been described in detail previously [18].

Pain assessment was documented in 55% (*n* = 447) of the cases, with higher assessment rates in PEN encounters. Both pain assessment and use of NRS were more frequent among younger patients and in cases with longer prehospital intervals, whereas use of NRS did not differ by EMS clinician competence. No differences were observed by sex. Initial pain intensity showed only minor variation, with older patients reporting slightly lower pain intensity than younger (Table 1).

**Table 1:** Pain assessment, initial pain intensity and analgesic treatment according to sex, age, clinician competence, and prehospital interval

	Sex			Age			EMS clinician competence			Prehospital interval		
	Female (n = 457)	Male (n = 359)	p	Younger (n = 413)	Older (n = 403)	p	PEN (n = 128)	Non-PEN (n = 677)	p	Shorter (n = 397)	Longer (n = 391)	p
Pain assessed, % (n)	52.1 (238)	58.2 (209)	0.089	63.9 (264)	45.4 (183)	<0.001*	67.2 (86)	52.7 (357)	0.003*	49.4 (196)	61.4 (240)	<0.001*
NRS documented, % (n)	38.9 (178)	44.8 (161)	0.100	49.4 (204)	33.5 (135)	<0.001*	50.0 (64)	40.3 (273)	0.050	36.3 (144)	47.8 (187)	0.001*
Initial pain intensity (NRS) (*n* = 339), median (IQR)	8 (6–9)	8 (6–9)	0.099	8 (6–9)	7 (5–8)	<0.001*	7 (5–8)	8 (6–9)	0.053	8 (6–9)	8 (6–9)	0.388
Analgesia administered, % (n)	39.4 (180)	37.6 (135)	0.613	47.9 (198)	29.0 (117)	<0.001*	50.0 (64)	36.5 (247)	0.005*	25.2 (100)	53.5 (209)	<0.001*
Analgesia administered for moderate–severe pain (*n* = 403), % (n)	63.6 (140)	59.3 (108)	0.410	65.6 (162)	55.5 (86)	0.046	80.6 (54)	57.7 (191)	<0.001*	46.2 (80)	74.1 (163)	<0.001*

Values reaching statistical significance, defined as *p* < 0.01, are marked with an asterisk (*)

Younger (≤64) and older (>64); median age 64 years

Shorter (≤38) and longer (>38); median prehospital interval 38 minutes

EMS = Emergency Medical Services; NRS = Numerical Rating Scale; PEN = Prehospital Emergency Nurse

Initial pain intensity was assessed among patients with documented NRS

Moderate–severe pain was defined based on documented NRS scores and clinical documentation, as described in the Methods

Column totals may vary because of missing data for EMS clinician competence and prehospital interval

Analgesia was administered in 39% (*n* = 315) of the cases, with higher proportions among younger patients, in cases with longer prehospital intervals, and in encounters involving PENs. Among patients with a documented pain assessment, 90% (*n* = 403) had moderate–severe pain and were therefore eligible for pharmacological treatment according to local guidelines. In this subgroup, treatment was more frequent, and higher rates were observed in encounters involving PENs and in cases with longer prehospital intervals (Table 1). Nonpharmacologic pain management measures were rarely documented (0.2%, *n* = 2), precluding further analysis.

Reassessment was infrequently documented overall. Any reassessment, defined as at least one additional documented pain assessment regardless of analgesia, was more common in encounters involving PENs and in cases with longer prehospital intervals. It was also more frequent among younger patients. No differences were observed by sex. When restricted to cases receiving analgesia, reassessment was more common among younger patients but did not differ by EMS clinician competence or prehospital interval at the predefined significance level. Mild pain at last assessment was observed in 10% (*n* = 84) of all cases and was more frequent in encounters involving PENs, whereas no significant differences were observed by sex, age, or prehospital interval (Table 2).

**Table 2:** Evaluation of treatment and pain outcome according to sex, age, clinician competence, and prehospital interval

	Sex			Age			EMS clinician competence			Prehospital interval		
	Female (n = 457)	Male (n = 359)	p	Younger (n = 413)	Older (n = 403)	p	PEN (n = 128)	Non-PEN (n = 677)	p	Shorter (n = 397)	Longer (n = 391)	p
Any reassessment, % (n)	18.2 (83)	21.2 (76)	0.287	27.4 (113)	11.4 (46)	<0.001*	31.3 (40)	17.4 (118)	<0.001*	12.6 (50)	27.9 (109)	<0.001*
Reassessment after analgesia (*n* = 315), % (n)	46.1 (83)	55.6 (75)	0.111	56.6 (112)	39.3 (46)	0.004*	62.5 (40)	47.4 (117)	0.036	49.0 (49)	52.2 (109)	0.628
Mild pain at last assessment, % (n)	8.5 (39)	12.5 (45)	0.064	9.9 (41)	10.7 (43)	0.731	22.7 (29)	8.1 (55)	<0.001*	8.1 (32)	12.8 (50)	0.035

Values reaching statistical significance, defined as *p* < 0.01, are marked with an asterisk (*)

Younger (≤64) and older (>64); median age 64 years

Shorter (≤38) and longer (>38); median prehospital interval 38 minutes

EMS = Emergency Medical Services; NRS = Numerical Rating Scale; PEN = Prehospital Emergency Nurse

Any reassessment refers to at least one additional pain assessment documented during the prehospital care episode, regardless of analgesia

Reassessment after analgesia refers to reassessment among patients receiving pharmacological analgesia

Mild pain at last assessment was based on the last documented pain assessment, including NRS scores and qualitative documentation, as described in the Methods

Column totals may vary because of missing data for EMS clinician competence and prehospital interval

## Discussion

In this cohort, encounters involving PENs showed higher rates of pain assessment, analgesia administration, and any documented reassessment, as well as a higher proportion of patients with mild pain at the last documented assessment. The difference in analgesia administration was particularly evident among patients with moderate–severe pain. Together, these findings suggest that EMS clinician competence may influence adherence to pain management recommendations and may also be reflected in documented end-of-encounter pain status.

Several mechanisms may underlie the observed differences. Formal specialist training has been shown to enhance clinician comfort with opioid administration, guideline adherence, and familiarity with multimodal analgesia [20]. In the prehospital field, increased pain assessment has previously been described in patients attended by advanced paramedics compared with EMS clinicians with lower levels of training [19]. In another study, analgesia was administered more often in physician-staffed response vehicles than in nurse-staffed units [3]. While previous studies on PEN competence are lacking, these findings suggest that clinician training, professional role, and decision-making autonomy may influence pain management practice in the prehospital setting. Targeted educational interventions may improve assessment and treatment practices in the short term, but such effects often diminish over time [27, 28]. Formal specialist competence, such as PEN training, may represent a more sustained integration of advanced clinical education into routine practice.

Differences in documentation practices may also have influenced the findings. Accurate documentation is an important component of EMS quality assurance, yet prehospital documentation is often incomplete [29]. As training and competence may improve adherence to structured documentation practices [30], the higher proportions of reassessments and mild pain in PEN encounters may partly reflect more consistent recording of repeated assessments rather than differences in clinical follow-up alone. Since documentation practices were not directly measured, this interpretation should be regarded as hypothesis-generating.

For patient age and prehospital interval, observed differences mainly concerned process measures, including pain assessment and analgesia administration. Older patients were less likely to receive documented assessment and treatment, consistent with previous reports of age-related disparities in prehospital pain management [3, 19]. Although differences in pain expression and communication may have influenced documentation, prior research suggests that such differences are unlikely to fully explain lower assessment rates among older patients [2]. More complex clinical conditions and comorbidity may instead shift attention toward diagnostics and stabilization rather than systematic pain documentation and treatment. Conversely, longer prehospital intervals showed higher proportions of assessment and treatment, in line with previous findings [13, 19], possibly reflecting more time and opportunity for structured care and documentation. However, these process differences did not translate into differences in mild pain at the last assessment. This measure reflects documented end-of-encounter pain status rather than measured change in pain intensity over time, and should therefore be interpreted as a proxy indicator of care quality rather than a measure of treatment effectiveness. The lower assessment and treatment rates among older patients warrant further research to determine whether they reflect patient presentation, clinical prioritization, documentation practices, or undertreatment.

Importantly, reassessment was documented in a small proportion of cases, limiting the number of observations available for evaluating changes in pain intensity and reducing the power to detect differences in documented pain outcomes. Although any reassessment was more frequently documented in PEN encounters, reassessment among patients receiving analgesia did not differ significantly by EMS clinician competence at the predefined significance level. More complete reassessment documentation would enable more robust analyses of changes in pain over time.

Overall, the findings underline the need to distinguish between documented care processes and patient-centered outcomes when evaluating prehospital pain management. Future studies should examine how assessment, treatment, and reassessment relate to patients’ pain trajectories, experiences, and clinical outcomes.

### Limitations

As a retrospective review of EPRs, this study is limited by its reliance on documented information, meaning that observed differences in pain management may reflect variations in documentation practices as well as actual differences in clinical care. For example, reassessment may have been performed but not recorded, leading to underestimation of reassessment frequency. The hierarchical approach allowed pain documentation to be captured more broadly than through standardized EPR fields and scales alone, but may also have introduced some degree of misclassification because free-text descriptions required interpretation. This risk may have been increased by the absence of abstractor blinding, independent assessment of coding consistency, and interrater reliability testing. However, data abstraction followed predefined variable definitions and coding rules, which were tested and refined before data collection to support consistency. The low proportion of documented reassessments limited the evaluation of changes in pain intensity and documented pain outcomes. Finally, the study was conducted in a single regional EMS system, which may limit generalizability.

Despite these limitations, the full EPR was reviewed for each case, enabling capture of pain assessments documented outside structured fields. The study also included a relatively large cohort of patients across an entire calendar year and evaluated multiple dimensions of pain management, including assessment, treatment, and reassessment.

## Conclusions

The PEN encounters showed higher rates of documented pain assessment, analgesia administration, and reassessment, with a higher proportion of patients having mild pain at the last documented assessment. Differences related to age and prehospital interval were mainly limited to process measures. Further research using designs that better support causal inference is needed to clarify whether clinician competence and care processes translate into improved pain relief.

## Data Availability

The datasets generated and analyzed during the current study are not publicly available due to restrictions related to patient privacy and the ethical approval but are available from the corresponding author on reasonable request and with relevant permissions. Original electronic patient records cannot be shared due to patient confidentiality.

## Abbreviations

EMS: Emergency Medical Services
NRS: Numerical Rating Scale
AAP: Acute Abdominal Pain
PEN: Prehospital Emergency Nurse
EPR: Electronic Patient Record
IQR: Interquartile Range
